# Agricultural land-use system management: Research progress and perspectives

**DOI:** 10.1016/j.fmre.2024.10.012

**Published:** 2024-11-04

**Authors:** Xiangzheng Deng, John Gibson, Malin Song, Zhihui Li, Ze Han, Fan Zhang, Wei Cheng

**Affiliations:** a Institute of Geographic Sciences and Natural Resources Research, Chinese Academy of Sciences, Beijing 100101, China; b College of Resources and Environment, University of Chinese Academy of Sciences, Beijing 100049, China; c Department of Economics, The University of Waikato, Hamilton, 3240 Waikato, New Zealand; d School of Statistics & Applied Mathematics, Anhui University of Finance and Economics, Bengbu 233030, China; e Beijing Technology and Business University, 100048 Beijing, China

**Keywords:** Land use optimization, Agricultural resource allocation, Agricultural carbon emissions and management, Land resource carrying capacity, Agricultural comprehensive production Capacity

## Abstract

The agricultural land-use system (ALUS) is a complex and coupled system centered on land use, which includes all human activities and their outcomes in utilizing agricultural land. With the emergence of issues such as climate change and food security, the land human coupling system has received widespread attention from scholars. Therefore, it is crucial to establish an ALUS management mechanism that sustainably utilizes resources, sequesters carbon, and reduces emissions while steadily improving food production. In this study, progresses are clarified in the theoretical framework and methods of ALUS management, including relevant research about agricultural land-use carbon emissions and management in the context of climate change, evaluation of the resource carrying capacity of a ALUS for food security, and layout optimization of agricultural land to improve land use capacity. It is crucial to fully leverage the role of ALUS in addressing climate change, ensuring nutrition and food security, providing ecosystem services, and promoting sustainable environmental development. However, the theoretical and empirical research on ALUS in China started relatively late. In this context, there is an urgent need to establish a better management system to accelerate the transformation of sustainable use of agricultural land in China, support high-quality development and ecological civilization.

## 1 Introduction

In recent decades, rapid human activities have caused serious environmental problems, and global environmental changes pose a serious threat to sustainable socio-economic development [1]. Especially the relationship between human activities and global warming has drawn widespread attention [2]. According to the report of the Intergovernmental Panel on Climate Change (IPCC), it is stated that greenhouse gas (GHG) emissions from human fossil-fuel use and land use change are the main causes of global warming, among which total GHG emissions from the food system were about one-third of the global anthropogenic total [3]. In this case, major developed countries in the world have gradually realized the pressure and challenge of GHG from the agricultural sector, especially non-carbon dioxide GHG emissions, to achieve global temperature control targets, and have begun to develop multi-scale integrated management and emission reduction solutions

An agricultural land-use system (ALUS) is a systematic, complex, and coupled system with land use at its core, and it includes all activities that humans engage in using agricultural land and their results. Thus, it is a great challenge to realize effective management of an ALUS. Recently, the ALUS in China has undergone profound changes. First, the unpromising international economic situation and the increasing contradiction between the supply and demand of agricultural products have aggravated the pressure to achieve a balanced increase in agricultural production. Second, it is increasingly urgent to implement ALUS management measures to adapt to and mitigate continuing climate change. Third, agricultural production is experiencing increasingly on resource and environmental constraints, e.g., declining quality of arable land and increasing pollution, which bring new pressure on ALUS. Fourth, agricultural science and technology innovation has provided new opportunities to promote high-quality development of agricultural industries, break through resource and environmental constraints, and achieve sustainable use of agricultural land. In this new situation, an ALUS that integrates adaptation to climate change and allows steady improvements in food production alongside sustainable use of resources, carbon sequestration, and emission reduction has become an inevitable choice. An ALUS is closely related to many disciplines that need to coordinate elements, such as forestry, agriculture, food, urban and rural areas, water and land resources, and climate change. Therefore, interdisciplinary and integrated research is urgently needed.

Case studies from various parts of the world underline the diverse approaches to ALUS management. For instance, implementation of integrated crop-livestock-forestry systems, showcasing significant improvements in soil health, productivity, and carbon sequestration in Brazil [4]. In Kenya, adoption of Climate-Smart Agriculture (CSA) among smallholder potato farmers has led to increased yields, improved resource use efficiency, and reduced environmental impacts [5]. In Netherlands, implementation of residents’ self-organization and the 51 % allocation of the area to agriculture has contributed to sustainable food supply in a city-region [6]. Empirical analyses focusing on the impacts of climate change on ALUS have elucidated several critical findings. There is strong evidence that rising temperatures, altered precipitation patterns, and increased frequency of extreme weather events are adversely affecting crop yields and food security globally [7]. Conversely, the implementation of CSA practices has been shown to mitigate some of these impacts, offering a pathway to enhance resilience and sustainability in ALUS.

China has entered a critical moment in accelerating rural revitalization and the comprehensive construction of a modern socialist country. It is also important to give full play to the role of its ALUS in coping with climate change, guaranteeing nutrition and food security, providing ecosystem services, and promoting sustainable environmental development. However, both theoretical and empirical studies of the ALUS in China began late. Against this background, there is an urgent need to establish a better management system to accelerate the transformation of sustainable agricultural land use in China and to support high-quality development and an ecological civilization. Given the complexity and diversity of the ALUS, a strong, active, and cooperative research group is required to conduct long-term academic research to meet the practical requirements of national environmental security.

**Fig. 1:**
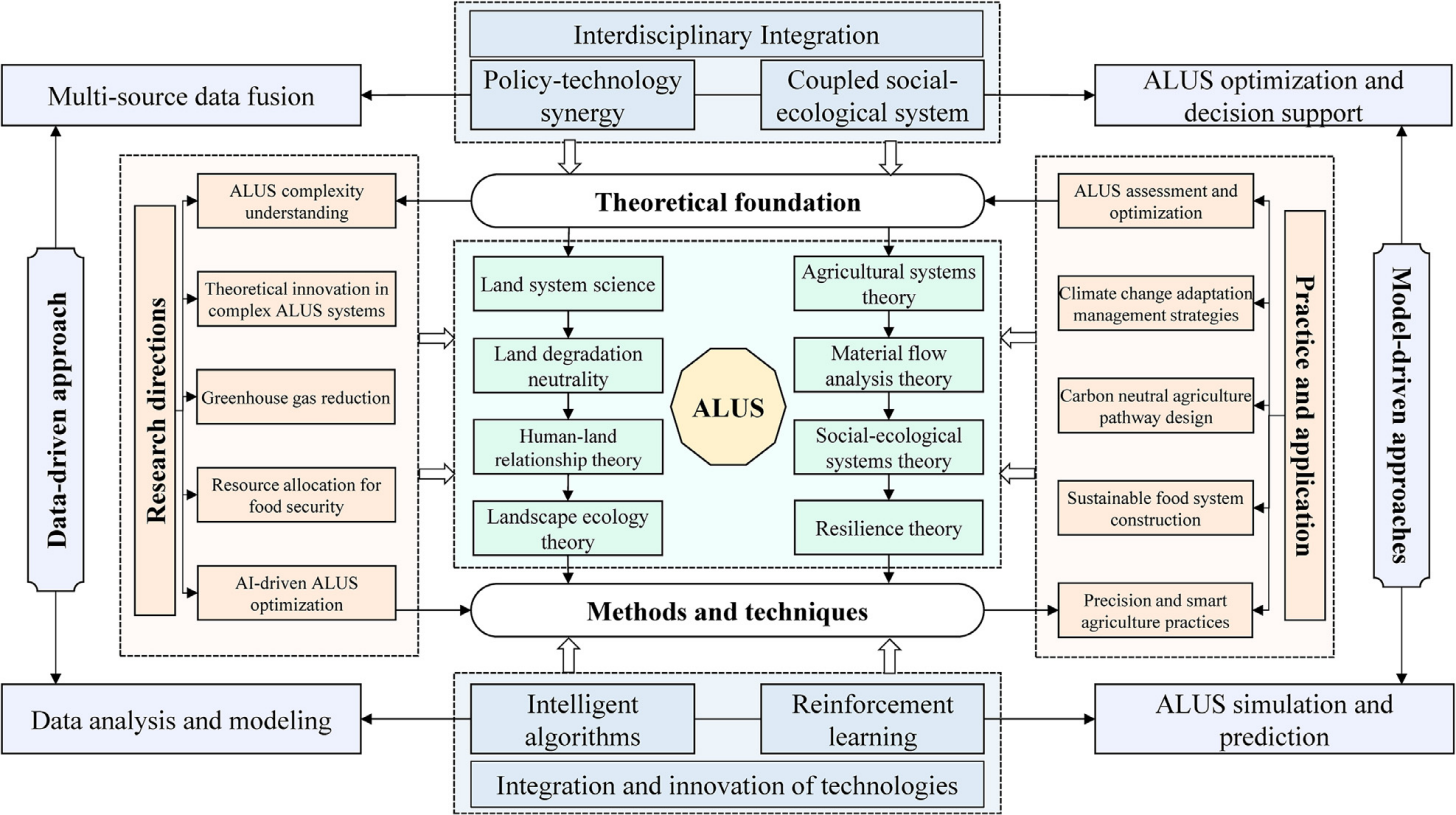
**Research Framework and Optimization Methods for Agricultural Land Use Systems (ALUS)**.

Therefore, this study will conduct in-depth summary and induction of the domestic and foreign research progress in ALUS management (Fig. 1), to provide support for scientific and rational use of agricultural land resources to achieve agricultural sustainable development. This study discusses the modeling methods and mechanism analysis of ALUS management. Then, taking climate change as the background, it explores the carbon emissions and management methods of agricultural land use. Further, it summarize the studies on the resource carrying capacity of agricultural land use for food security. Finally, it explores the layout and optimization status of agricultural land use capacity improvement. The goal of this study is to provide a clear understanding of the current situation and hot topics of ALUS management through the above discussion, and to assist in the sustainable development of agricultural land.

## 2 Modeling approach and mechanism analysis of agricultural land use system management

The changes in land have had irreversible impacts on society and the environment, ranging in scale from decades to hundreds of years [[8], [9]]. Land has both social and natural attributes. In many cases, land cover and land use have not changed, but the functions and effects of their land systems have undergone significant changes. For example, changes in grassland types can affect regional carbon storage, and changes in cultivated land quality can affect food production [10]. In the Amazon rainforest, extensive deforestation leads to the release of a large amount of carbon stored in vegetation and soil into the atmosphere as carbon dioxide, significantly increasing regional and global carbon emissions. Conversely, large-scale afforestation projects help absorb carbon dioxide from the atmosphere, increasing the region's carbon storage capacity. These changes clearly demonstrate the significant impact of changes in agricultural or forested areas on regional carbon storage [11]. Based on land cover and land use, the land use system had been proposed, which is defined as a combination of land ecosystem and land economic system connected by human activities in a certain area. Specifically, it includes the interaction and influence of subsystems such as population, land resources, land environment, economy, social technology, and management regulation within the region, forming an organic whole with certain functions [12]. From a macro perspective, ALUS is a combination of land and agricultural systems, and is a result of some activities that humans engage in using cultivated land. Agricultural land provides a place and resource supply for human activities. It is very important to scientifically utilize land use system theory and methods to guide ALUS management practices and improve farmland productivity through sustainable farmland use and agricultural technology innovation. The research on ALUS management modeling methods and mechanism analysis mainly includes theoretical innovation of institutions, construction of method systems, and mechanism analysis. The research findings can provide guidance for decision-makers, promoting effective land use management, sustainable agricultural practices, and strategic formulation that comprehensively considers productivity, environmental protection, and socio-economic development.

### 2.1 Global agricultural land-use system management: A comparative analysis

The global management of agricultural land use is complex and diverse, influenced by the resource endowments, economic conditions, and policy orientations of various countries and regions [13]. The United States, with its vast agricultural lands, employs highly mechanized and industrialized production methods, supported by government subsidies and ecological protection measures [14]. In contrast, China practices intensive farming and technological innovation, optimizing resource allocation through land policy adjustments [15]. Europe's agricultural models are diverse, emphasizing environmental protection and sustainable development, with a common agricultural policy to guide this effort [16]. Africa, despite its abundant land resources, has lower productivity, which it seeks to improve through international cooperation aimed at boosting agricultural technology and productivity [17]. Major agricultural countries employ diverse policy frameworks and land ownership systems to manage agricultural land use, support farmers, and balance agricultural development with environmental protection and sustainability.

Major agricultural countries employ diverse policy frameworks and land ownership systems to manage agricultural land use and support farmers. China utilizes a collective land ownership system, ensuring farmers' interests and agricultural development through land contracting and state subsidies [18]. Conversely, the United States predominantly has private land ownership, supporting farmers with agricultural conservation contracts and subsidy systems to promote environmental protection [19]. Brazil balances economic development with ecological conservation through agricultural extension laws and environmental regulations [20]. India supports small farmers by promoting high-yield crops and irrigation technologies, continuing the legacy of the Green Revolution to enhance productivity and rural livelihoods [21]. The EU provides subsidies through the Common Agricultural Policy (CAP), with a focus on environmental protection and land planning to ensure sustainable agricultural practices [22]. These policy approaches reflect each country's unique socio-economic conditions and priorities in managing agricultural land use and supporting farmers.

Technological application plays a crucial role in enhancing agricultural productivity and sustainability in major agricultural countries. China promotes smart agriculture, high-yield crops, and rural e-commerce to enhance farmers' market access and information [23]. The United States employs precision agriculture and biotechnology to achieve high mechanization, optimizing resource use and minimizing environmental impacts [24]. Brazil focuses on bioenergy and agricultural mechanization, using remote sensing to improve land use planning and management [25]. India promotes low-cost technology and agricultural information services, combining traditional and modern technologies to support small farmers and enhance their resilience [26]. The EU develops environmental technologies, data platforms, and renewable energy to promote sustainable agricultural practices and reduce greenhouse gas emissions [27]. These innovations highlight each country's efforts to modernize agriculture while addressing sustainability challenges.

Sustainable measures are essential for ensuring the long-term viability of agricultural systems. China advances ecological agriculture, soil protection, and emission reductions to mitigate the environmental impacts of intensive farming [23]. The United States employs conservation reserve programs and crop rotation to promote soil health and biodiversity conservation [19]. Brazil uses sustainable soybean programs and agricultural ecological zone management to prevent deforestation and protect sensitive ecosystems [25]. India supports organic agriculture and traditional knowledge, implementing soil and water conservation practices to enhance the resilience of small-scale farming systems [21]. The EU enforces green policies and promotes cross-border cooperation and biodiversity protection to tackle transboundary environmental challenges [27]. These sustainable measures reflect each country's commitment to balancing agricultural production with environmental stewardship, considering their specific ecological and socio-economic contexts. By adopting various sustainable practices and policies, major agricultural countries aim to ensure the long-term sustainability of their agricultural systems and the well-being of their farmers and communities.

### 2.2 Theoretical innovation in agricultural land use system management

#### 2.2.1 Review of existing theoretical frameworks

Relevant theories have been applied to land use systems and have had a more profound impact on ALUS management. It mainly includes political ecology theory, ecological footprint theory, resilience theory, and human-land relationship theory. For example, to manage complex agro-socio-ecological requirements, Flachs and Richards (2018) applied political ecology theory to explore the relationship between environmental issues and political [28], economic and social factors, and argues for a dialectical view of the relationship between political ecology and economy in ALUS. It provided ideas for shaping and implementing land-use policies. Ecological footprint theory can simplify complex ecological processes and interactions by focusing on footprint indicators. Zuazo et al. (2011) emphasized that the sustainable ALUS requires attention to spatial layout options for economic activities [29], validating the ineffectiveness of the ecological footprint approach for public policy. The resilience theory, which can deal with the complexity and interconnectedness of socio-ecological systems, is a commonly used approach in the field of ALUS construction and helps to enhance long-term sustainability by building the system's resistance to perturbations. Human-land relationship theory focuses around the interactions between human society and the natural environment, providing a balanced view of land-use impacts and emphasizing the long-term consequences of ALUS management. VanWey et al. [30] comparatively analyzed theories related to land-use change, recognizing that coordinated development of people and the land is an important aspect that must be considered in the management of ALUS.

#### 2.2.2 Identifying gaps and contradictions in existing theories

Existing theories mainly target sustainable development, considering multiple dimensions within the ALUS, including social, economic, political, and environmental aspects, aiding in a comprehensive understanding and resolution of ALUS issues. However, existing theories on ALUS still have some drawbacks. Firstly, the theories are complex and challenging to implement. These theories often involve multifaceted factors and intricate interactive relationships, requiring interdisciplinary cooperation and complex data analysis beyond a certain scope, making comprehensive control difficult. Secondly, the effective application of relevant theories demands a large amount of high-quality data and resources, which may be challenging to achieve in regions with limited economic and technical resources. Additionally, some theories may require expensive detection and analysis tools for application, incurring high costs. Thirdly, some theories may struggle to manage the complexity of agricultural land systems in specific applications, possibly ignoring local and cultural differences, resulting in a disconnect between theory and practice. Fourthly, due to regional heterogeneity, there exists a certain distance between policies and practices. Although relevant theories provide rich analytical tools and strategies, failing to consider practical issues like the actual policy environment may limit their application and dissemination in China. Meanwhile, facing global major issues such as climate warming, balance of food supply and demand, agricultural carbon reduction, there is still a need for a more systematic, comprehensive, and specific ALUS to guide practice.

Accurate simulation of land system dynamics is the premise of analyzing the dynamic evolution of land system and analyzing its effect. The early classical land simulation model determined the conversion rules through the self-adaptation between land categories, and the “bottom-up” conversion rules should reflect the global and orderly pattern generated by the local individual behavior of a complex system [31], such as the Conversion of Land Use and its Effects (CLUE) model, Slope Land use Exclusion Urban extent Transportation Hill shade (SLEUTH) model, and Cellular Automaton-Markov (CA-Markov) model. Although these models can greatly restore the real dynamic transformation of land system, it is difficult to analyze the impact of social system and macro policies on land use decision-making behavior and its results by ignoring the influence of human choices or decision-making behaviors. Chen and Wang (2009) constructed models for individual farmers, interactions between similar farmers, and the impact of the market on farmers based on the Belief, Desire, Intention (BDI) structure generated by decision-making [32]. Liu et al. [31] considered the planning behavior of the government and the housing selection behavior of urban residents, and the government planned land resources based on the allocation theory of environmental economic resources and the concept of sustainable development. Deng (2011) proposed that land has multiple suitability and heterogeneity, and drew a competition curve that responds to changes in land demand in agricultural and other industrial sectors and drives the conversion between different land use types [33]. This made up for the limitations of traditional land system dynamics mechanism analysis in examining and expressing land element attributes, and systematically elaborated and quantitatively expressed the characteristics of land multiple suitability and heterogeneity. The “human - land” relationship is the core idea of land dynamic system simulation, and further consideration is needed on how to more reasonably incorporate the influence of “people” into the model construction.

#### 2.2.3 Our theoretical framework: structure and innovation

The allocation and utilization system of land and water resources in regional agricultural production is a complex system that includes multiple scales and elements. How to use a theoretically complete method to achieve the cross scale organic combination of macro scale pattern information and micro scale process information, and solve the error problem, multi-scale problem, and large memory demand problem in modeling the Earth's surface layer, is an important challenge to improve the high-precision spatiotemporal allocation and utilization of key resources in agricultural systems. High precision surface modeling enables the grid representation of surface systems and ecological environment elements [34]. However, due to its high accuracy, there are problems such as large data volume, high computational costs, and slow processing speed, making it difficult to apply to large-scale modeling of the Earth's surface. To this end, we adopt an interdisciplinary approach based on perspectives of climate change and food security, serving food security strategies and the “carbon peaking and carbon neutrality” goals, aiming to establish a sustainable ALUS that utilize resources efficiently, reduce emissions, and steadily increase food production [35]. Through clarifying the complexity of ALUS, we aim to control the specificity of the system, focusing on improving agricultural land carbon emissions and management under the backdrop of climate change, evaluating and regulating the comprehensive effects of ALUS on food security and resource carrying capacity. This effort seeks to integrate regional characteristics with general principles, forming a theoretical framework and management practices for ALUS that align with the socio-economic, ecological, and cultural needs of China.

### 2.3 Classic examples of model-informed practices

Brazil's transition was driven by the need to combat the negative impacts of traditional agriculture, such as soil erosion, decreased soil fertility, and high carbon emissions, which significantly threatened its vast agricultural landscapes, including the cerrado and the Amazon regions [36]. The adoption of conservation agriculture practices resulted in remarkable increases in soil carbon stocks, a crucial factor in climate change mitigation. No-till farming, by reducing soil disturbance, helps in retaining carbon in the soil, preventing its release into the atmosphere as carbon dioxide. This method, combined with cover cropping and crop rotation, has enhanced soil structure and health, leading to improved water retention, reduced soil erosion, and minimized the need for chemical fertilizers.

China's terracing and agroforestry program effectively combats soil erosion and conserves water. By converting steep slopes into terraced fields and strategically planting trees and shrubs, this program significantly decreases water runoff velocity, soil erosion, and nutrient loss while improving water infiltration and retention. Terracing safeguards topsoil, enhances soil moisture, and boosts agricultural productivity. Integrated trees and shrubs stabilize terraces, reduce erosion, promote biodiversity, and offer additional income through fruit, nut, and timber production. The program's impact on the Loess Plateau is remarkable, with improved microclimates, moderated temperatures, enhanced precipitation infiltration, and reduced evaporation losses [37]. Furthermore, the increased vegetation cover contributes to carbon sequestration, playing a crucial role in climate change mitigation. Overall, this holistic program ensures a stable and fertile environment for agriculture while delivering broader environmental benefits, making it essential for sustainable land management and ecosystem preservation.

### 2.4 Analysis of implemented policies

The European Union's Common Agricultural Policy (CAP) stands out due to its wide-ranging influence on agricultural practices, environmental sustainability, food security, and socio-economic development within the member states [38]. On the positive side, the CAP has contributed significantly to stabilizing food prices, ensuring a consistent food supply, and supporting farmers’ incomes across Europe, thereby addressing a core pillar of food security. Moreover, recent reforms have increasingly focused on integrating environmental sustainability into the policy framework, by incentivizing practices such as crop diversification, maintenance of permanent pasture, ecological set-aside areas, and agri-environmental schemes, which aim to enhance biodiversity and manage natural resources more efficiently. However, areas for improvement remain evident. Critics argue that the CAP has historically favored larger farming operations through its subsidy structures, potentially marginalizing smaller farms and exacerbating socio-economic disparities in rural areas. Additionally, despite the greening measures, environmental organizations express concerns that the CAP does not go far enough in reducing agriculture's carbon footprint or reversing biodiversity loss, citing the need for more robust environmental criteria and stricter enforcement.

Turning to a different context, Brazil's Forest Code is another landmark policy with profound implications for agricultural land use [39]. It mandates the preservation of a percentage of land for native vegetation on private farms, aiming to curb deforestation rates which have been direly high, especially in the Amazon rainforest. The Forest Code represents a pioneering attempt to balance agricultural development with environmental conservation. Successes include the establishment of legal mechanisms for land registries, which improve land governance and the potential for reforestation in deforested areas. Nonetheless, enforcement challenges persist, driven by inadequate monitoring and control mechanisms, coupled with continual pressures from agricultural expansion. These limitations highlight the persistent struggle to achieve a practical balance between agricultural productivity and environmental stewardship in regions of high biodiversity and global environmental significance.

### 2.5 Methodological system of agricultural land use system management

The management of the ALUS in China, while historically effective in certain aspects, faces challenges in terms of integrating modern theoretical frameworks and advanced technical systems to address the evolving needs of sustainable agriculture. In response to this problem, researchers have made numerous advances in the construction of technical systems for simulation and optimal management analysis of the regional ALUS (Table 1). From the perspective of research methods, statistical analysis based on expert experience or social surveys, numerical analysis based on statistical data, and spatiotemporal change detection based on multi-source remote sensing information have emerged. These methods result in many land system response simulation models based on spatial models, making more and more scholars pay attention to the corresponding mechanisms of land systems. In the 1990s, the United States Agricultural Center used remote sensing data to draw crop distribution maps, marking the beginning of the application of remote sensing technology in agricultural land management. Scholars have constructed various time series indices based on the sensitivity of different crops to different combinations of sensors, such as using seasonal Enhanced Vegetation Index (EVI) to analyze regional replanting, using Normalized Differential Vegetation Index (NDVI) time series data to predict crop yields, and combining NDVI and phenological indicators to draw abandoned agricultural maps. The combination of multi temporal, multi sensor, and multi resolution remote sensing data with geographic information technology can enhance data collection, modeling, and decision support capabilities, and is also used to evaluate large-scale agricultural land and crop expansion patterns [[40], [41]]. Spatial models are mainly used to explore the spatial patterns of land and crops, such as simulating the potential development space of crops on a global scale, and predicting the spatial patterns of crops under different climate impacts.

**Table 1:** **Comparative analysis of methodological approaches in ALUS research**.

Model systems	Approaches	Applications
Spatial geographic modeling	Land productivity assessment, land system response modeling	Exploring land spatial patterns and modeling potential crop development space
Economic model	Efficiency assessment, econometric analysis	Analyzing land use efficiency and optimizing management of ALUS
Multi-scale resource management policy assessment model	Coupled analysis, general equilibrium analysis	Exploring the policy impacts on resource utilization and provide parameters for coupled regulation of land and water resources
Multi-scale watershed water-socio-economic model	Regional input-output analysis, ecohydrological process modeling	Regulating ecohydrological processes to optimize the integrated use of land and water resources
Climate change-crop phenology-resource utilization feedback analysis model	Attribution analysis of phenological changes, risk assessment of agricultural production, parameterization of surface cover changes	Exploring changes in agricultural production and attribution effects to enhance regional climate modeling capabilities

The research focus of the model is to capture the complex relationships between multiple variables, reveal the basic mechanisms driving land use decision-making, and then simulate and predict the development of ALUS [[35], [36], [37], [38], [39], [40], [41], [42], [43]]. According to the different influencing factors of agricultural land use changes, models are usually divided into geographic models and economic models [44]. At present, land use efficiency and benefit assessments represented by traditional economic models are difficult to effectively reflect complex macro natural and resource environmental processes [45]. The evaluation model led by spatial geographic information technology is difficult to reveal the logical relationship between socio-economic and human factors [46]. How to leverage the advantages of multi-model coupling in land use processes and establish technical methods with practical capabilities to enhance land economic, technological, and ecological efficiency assessment has always been a scientific issue that has attracted attention from peers both domestically and internationally. Polimeni (2005) used a comprehensive framework consisting of econometric model, Geographic Information Systems (GIS), and Monte Carlo model to predict the impact of residential development on agricultural landscapes in the Hudson Valley basin of Darcy County, New York [47]. Lovett (2009) combined empirical model with GIS, fully considering agricultural land quality and the distribution of currently planted food crops, generated yield maps and estimated regional energy production potential [48]. Deng et al. [49] introduced efficiency theory into agricultural land management, developed a multi-scale and multi factor efficiency evaluation and optimization management model for land use, improved the ability to evaluate land economic, technological, and ecological efficiency, and solved the effective combination problem of multi-scale land use efficiency evaluation and optimization management. The above research has enhanced the allocation of land use efficiency and optimized management methods.

The development of agriculture is centered around the coupling of land and water resources, and the efficient coupling utilization of land and water resources is an important field of efficient agricultural development. Some scholars have attempted to scientifically construct theoretical research and models for multi-scale land and water resources management policy evaluation in agricultural systems. Bithell and Brasington (2009) established a coupled feedback model between crop growth, land evolution, and hydrological cycles, evaluating the impact mechanisms of agricultural and hydrological changes [50]. Wu et al. [51] constructed a comprehensive theoretical model with the goal of improving the comprehensive utilization efficiency of land and water resources in river basins, and quantitatively evaluated the performance of regional industrial structure optimization and adjustment in improving the utilization efficiency of land and water resources. Han et al. [52] explored the impact mechanism of shallow groundwater on land management for cotton cultivation using the Hydras-1D model based on the cycling process of the soil plant atmosphere system. Zellner (2020) established a Hydroman model that couples human and hydrological processes and validated it with observed groundwater level patterns and crop yield data [53]. The research on the coupling models of land and water provides parameters for the coupling regulation and management of land and water resources and proposes operational suggestions for land and water resource management, systems, and policies to achieve resource conservation and efficient utilization.

Comprehensively, researchers have made remarkable progress in recent years in regional ALUS simulation and optimization of the management and analysis technology system. Through statistical-based numerical analysis, detection of spatial and temporal relationships and other methods, a spatial model-based land system response simulation model has been constructed, revealing the mechanism of land use change. The application of remote sensing technology in farmland management has further enhanced data collection, modeling and decision support capabilities. The combination of spatial geographic models and traditional economics models improves the land spatial pattern simulation and land use efficiency assessment studies, and enhances the ability to assess the economic, technical and ecological benefits of land. The coupled model of land and water resources quantitatively evaluates the impact of regional policy changes on the utilization efficiency of land and water resources through socio-economic coupling analysis and policy impact assessment, providing a scientific basis for the efficient management of land and water resources. The Climate Change-Crop Climate-Resource Utilization Feedback Analysis Model provides spatial parameterization schemes for biophysical and anthropogenic management measures of large-scale crop models, which provides a decision-making tool for regional agricultural production to adapt to climate change. The integrated application of the above model systems enables ALUS to achieve sustainable use and optimal allocation of resources in a more scientific and efficient manner.

### 2.6 Comparative review of global IAMs and china's land system models

The GCAM model evaluates interactions between the economy, energy, and climate, providing insights for climate change mitigation and adaptation in agriculture. It covers global activities like land use, aiding in forecasting impacts on food production and environmental integrity. Conversely, GTAP focuses on economic transactions and trade flows, analyzing trade policies' effects on agriculture globally. Despite their differences, both models are vital for informed decision-making in the nexus of agriculture, economics, and environmental sustainability, highlighting the importance of IAMs in managing global agricultural and land-use challenges.

These models vary in foundation, methodology, and scope. They highlight areas for enhancing China's land system models' sophistication and robustness, while also acknowledging the unique contributions of Chinese models, especially in capturing detailed dynamics of China's agricultural and land-use changes. Global models like GCAM provide broad insights but may lack local detail, which Chinese models cover well due to their localized data and specific methodologies. However, Chinese models could improve by incorporating the advanced scenario analysis and global perspective of international IAMs, enhancing their ability to address transboundary impacts.

Building on the insights gleaned from the comparative review between global IAMs and China's land system models, we propose a suite of targeted improvement strategies aimed at advancing the sophistication and utility of China's models. A pivotal recommendation involves the integration of broader socio-economic factors into the modeling framework. By incorporating variables such as demographic changes, urbanization trends, and socio-economic development scenarios, China's models could offer more nuanced and comprehensive insights into land-use dynamics. Furthermore, we advocate for an enhancement of scenario analysis capabilities, enabling the exploration of a wider array of future pathways and their potential impacts on land use and sustainability outcomes. This could involve adopting the sophisticated scenario development and analysis techniques employed by models like GCAM, which facilitate the examination of complex interactions and feedback mechanisms between climate policies, energy use, and agricultural production systems. Additionally, adopting more flexible and scalable computational frameworks, akin to those used in GTAP, could significantly improve the models' ability to handle large datasets and conduct granular, region-specific analyses. These improvements, inspired by the strengths of global IAMs, would not only refine the robustness of China's land system models but also enhance their applicability in supporting policy-making processes for sustainable land use and environmental conservation.

The GTAP model, with its focus on global trade dynamics, is particularly relevant for China due to its significant role in international trade and the resulting effects on domestic land use and agriculture. Additionally, the GCAM model's ability to simulate the impacts of international climate policies provides essential insights into potential future scenarios for China, aiding in the development of resilient land-use strategies. Incorporating these global perspectives into China's land-use models could greatly enhance their detail, scope, and relevance, thereby improving the tools available to policymakers for navigating sustainable development and land management challenges.

### 2.7 Mechanistic analysis of agricultural land use system management

The management of regional agricultural land use systems faces the problem of collaborative competition with external systems such as urban systems, while also encompassing the mutual feedback between internal climate systems, resource utilization systems, agricultural production systems, and other systems. Effectively analyzing the interaction mechanism between internal and external multiple factors and systems in agricultural systems is the foundation for achieving optimal management of regional agricultural land use systems [54]. The scarcity of land resource, the diversity of land use, and the competition for land rights and interests have led to a series of land space problems such as urban-rural development imbalance, ecological environment deterioration, and inefficient land use. Among them, the competition and coordination issues of agriculture, towns, and ecological spaces have attracted much attention [[55], [56], [57]]. In the study of external impacts, Fazal (2001) explored the impact of urban development on agricultural land in the city of Saharanpur, indicating that non construction development in the city caused the greatest losses [58]. Deng et al. [[59], [60]] discovered for the first time through empirical research the marginalization characteristics and regional differentiation patterns of urbanization occupying arable land, and used a multi-level econometric model to analyze the influencing factors and mechanisms of urban expansion occupying arable land. Yu (2022) considered the expansion of the high-speed rail network, which had an indirect impact on farmland acquisition and a direct impact on the agricultural land industry [61]. In the internal impact study, Jin et al. [62] established a system dynamics model based on subsystems of capital input output, land use, agricultural production, and social impact. Fang et al. [63] quantitatively evaluated the production, ecological, and social security functions of cultivated land use, and explored the spatial differentiation and regional coordination of the multifunctional balance relationship of cultivated land use. Li et al. [64] analyzed the interaction mechanism between the natural, social, and economic subsystems of agricultural land intensification. The exploration of complex impact mechanisms of multiple factors in external and internal systems has promoted the research of innovative analysis theories and methods for land use balance and optimization, providing decision-making reference information for the coordination and balance of land use conflicts under the integration of national spatial planning systems.

The mutual feedback mechanism between climate change, crop phenology, land and water resource utilization, and agricultural production in regional agricultural systems is also one of the basis for constructing the model system. The complexity of the agricultural system determines the complex interaction between multiple factors such as water, land, and climate. Quantitative analysis of the connections between multiple factors and processes is an important support for optimizing regional agricultural production and ensuring food security. Scholars have conducted extensive mechanism analysis and simulation work on the interaction between subsystems such as climate change, land use, and agricultural production in regional agricultural systems. Tao et al. (2006) demonstrated the necessity of temperature changes changing crop phenology, thereby affecting crop yield, and the comprehensive impact of temperature and carbon dioxide concentration on the physiological processes and mechanisms of crop growth and production [65]. Shi et al. [[66], [67]] revealed the feedback effects and mechanisms of crop planting methods, agricultural irrigation, and agroforestry grassland conversion on regional climate change through simulation research and mechanism diagnosis of the feedback effects of agricultural land use on climate change in northern China. Fróna et al. [68] explored the connection between climate change, population growth, and agricultural production from a theoretical perspective. Although the above research has to some extent explored the impact mechanism of multiple factors on agricultural systems, there is still a large gap in quantitative and systematic research.

Previous research outlines practical implications and case studies from reviewed studies aimed at guiding policymakers, practitioners, and stakeholders in sustainable agricultural land use (Table 2). Policymakers are urged to create policies that enhance soil health, promote water conservation, and support sustainable crop rotation. They are also encouraged to integrate climate resilience into policies and foster agro-ecological methods, balancing environmental protection with food production and economic impacts. Innovations such as carbon credit systems and agri-tech zones are also emphasized. Practitioners are advised to adopt practices like no-till farming, drip irrigation, and diverse crop rotations. Advanced techniques including precision farming and IoT monitoring are recommended to improve soil health, decrease reliance on chemicals, and boost economic returns. The adoption of renewable energy and digital agriculture platforms is encouraged. Stakeholders should participate in collaborative research and community-led initiatives focusing on pest management and water conservation, and promote technology sharing and food waste reduction. The importance of engaging stakeholders in policy making and the influence of community actions on local agriculture are highlighted. Case studies from around the globe illustrate successful sustainable practices, such as Vietnam's integrated shrimp and mangrove farming, Kenya's solar irrigation projects, and Brazil's cover cropping for soil improvement, showcasing varied and innovative sustainable agricultural strategies.

**Table 2:** **Practical implications and case studies of the reviewed studies for policymakers, practitioners, and stakeholders**.

Categories	Implications	Guidance	Insights	Directions
Policymakers	1. Designing policies for soil health improvement. 2. Incentive structures for water-saving technologies. 3. Regulations for sustainable crop rotations.	1. Integrating climate resilience in agricultural policies. 2. Support for agro-ecological approaches. 3. Policies for reducing agricultural emissions.	1. Balancing food production with environmental protection. 2. Economic impacts of sustainable farming. 3. Long-term benefits of agro-biodiversity.	1. Innovating policies for carbon credit systems. 2. Development of agri-tech zones. 3. Focus on sustainable food systems.
Practitioners	1. Adoption of no-till farming. 2. Utilization of drip irrigation. 3. Implementing crop rotation and diversification.	1. Precision farming techniques. 2. Using IoT for soil and weather monitoring. 3. Engaging in community-supported agriculture (CSA).	1. Economic advantages of sustainable practices. 2. Enhancing soil health and productivity. 3. Reducing dependency on chemical inputs.	1. Expanding use of renewable energy in farming. 2. Adopting regenerative agriculture. 3. Embracing digital agricultural platforms.
Stakeholders	1. Collaborative research on pest management. 2. Community initiatives for water conservation. 3. Workshops on sustainable farming techniques.	1. Partnerships for technology sharing. 2. Initiatives for food waste reduction. 3. Advocacy campaigns for climate-smart agriculture.	1. Importance of stakeholder engagement in policy formulation. 2. Role of NGOs in promoting agroecology. 3. Community impact on local agricultural practices.	1. Developing platforms for knowledge exchange. 2. Supporting local farmer markets. 3. Investing in agricultural education programs.
Case Studies	1. Vietnam's integration of shrimp farming with mangrove forests. 2. Kenya's solar-powered irrigation projects. 3. Brazil's use of cover cropping to improve soil health.	1. India's digital agriculture initiatives. 2. The Netherlands' precision farming. 3. USA's conservation agriculture practices.	1. China's rice-fish co-culture for sustainability. 2. Australia's drought-resistant crop research. 3. Cuba's organic urban farming movement.	1. Colombia's community-based deforestation prevention. 2. Israel's water-saving drip irrigation technologies. 3. Germany's renewable energy in agriculture

## 3 Agricultural land use carbon emissions and management in the context of climate change adaptation

Agricultural land system delineates the accounting boundary for our examination of carbon emissions, centering primarily on CO_2_ emissions stemming from fossil fuel combustion, land-use changes, and industrial processes. This delineation aligns with the most significant sources of carbon emissions pertinent to our research objectives and adheres to the Intergovernmental Panel on Climate Change (IPCC) guidelines. While our primary focus is on carbon dioxide, the scope of our analysis also includes non-carbon greenhouse gases (GHGs) such as methane (CH_4_) and nitrous oxide (N_2_O), recognizing their critical role in global warming and climate change. Despite the predominant emphasis on CO_2_, incorporating data on CH_4_ and N_2_O is crucial given their higher global warming potential (GWP) over a 100-year period compared to CO_2_, which provides a more holistic view of the total impact of greenhouse gas emissions.

The 2030 carbon peak and 2060 carbon-neutral targets (called the “dual carbon” goals) are major strategic deployments made by the Party Central Committee after careful consideration and a solemn commitment along with the rest of the world to address climate change. To achieve these goals, extensive and profound systemic changes in the current socioeconomic system will be required. Agricultural land use is an important factor that affects regional carbon emissions, and plantations and livestock related to agricultural land use are important sources of carbon emissions. Major developed countries have gradually realized the pressure and challenges that carbon and non-carbon greenhouse gas emissions from the agricultural sector place on global temperature control targets and have started to develop countermeasures. Current policies and studies addressing climate change generally lack quantification of indicators for carbon and non-carbon GHG emissions, and there is controversy over the uncertainty of the future reduction potential of CO_2_. Some existing research points to the major role of the industrial sector in carbon emission reduction, but there is a lack of comprehensive and systematic research on emission efficiency, emission reduction measures, emission control costs, and the potential impacts of key agricultural land uses. Carbon emission and management research from the perspective of agricultural land use can provide insight into the mechanisms influencing the regional carbon cycle and can comprehensively guide the low-carbon development of the economy and society in the fields of land system planning, industrial structure regulation, land development and improvement, and urban and rural construction. The past studies accumulated a strong foundational advantage in agricultural land use and management research and has achieved fruitful results in the fields of land system economic theory innovation [[69], [70], [71]], analysis of the driving mechanisms of land use system change, land system use transformation, and agricultural land use carbon emission assessment. On this basis, integrating the advantages of management, geography, economics, environmental science, and other disciplines, combined ALUS and carbon emission management, which broadened the research scope, achieved a series of outstanding results in land use carbon emission effects, agricultural land use carbon emission accounting, and low-carbon land use optimization and management, among other things.

### 3.1 Agricultural land use carbon emission/reduction index construction and model coupling

The multi-model coupling process and mechanism analysis are the core contents of ALUS research. Multiscale data collection, parameter preparation, index construction, and multi-model coupling analysis of agricultural land use carbon emissions serve to improve the efficiency of agricultural land use carbon emission systems and the transformation of agricultural ecological civilization. Bai et al. (2018) and Wang et al. (2019) have conducted technical research to address the current difficulties in accurately assessing the development and management of agricultural land use carbon emissions and achieved a technical breakthrough in the spatially explicit assessment of carbon emission efficiency and emission reduction potential [[72], [73]]. This breakthrough is relevant to regional agricultural land use under the strategy of rural revitalization, and involved updating the efficiency evaluation indexes from those that were focused on quality assurance and efficiency to instead allow for economic efficiency-oriented, ecological efficiency-led, and other efficiency-balanced indexes. The breakthrough also improved the carbon emission reduction index system to serve the realization of the “dual carbon” goals and the construction of an ecological civilization.

In the realm of the constructing data/parameters and indicators for land use carbon emissions and reduction, current research mainly focuses on the collection of multi-scale data, the preparation of parameters, the construction of indicators, and the multi-model coupling analysis of agricultural land use carbon emissions. These endeavors aim to improve the efficiency of agricultural land use carbon emission system and facilitate the transition towards agricultural ecological civilization [[74], [75], [76]]. However, a significant challenge persists in accurately assessing the efficiency of agricultural land-use carbon emissions, hindering the evaluation and management of agricultural carbon emissions development. There is a need for breakthroughs in the technology of explicit evaluation of carbon emission efficiency and mitigation potential in the spatial domain.

At present, scholars have collected the existing global CO_2_ data from carbon satellite, ground-based CO_2_ observations, CO_2_ driving fields from CMIP6 model, and anthropogenic carbon emission data. In global sea-land-atmosphere carbon cycle coupling models, these datasets are incorporated to investigate the corresponding relationship between global atmospheric CO_2_ concentrations and surface warming processes [[77], [78], [79]]. Typical regions exhibiting changes in global CO_2_ concentrations and surface temperatures are selected, allowing for the analysis of the climate effects and response mechanisms of dynamic atmospheric CO_2_ concentration distributions [80]. Sensitivity curves depicting the relationship between atmospheric CO_2_ concentration and surface temperature are generated, enabling a quantitative assessment of the impact mechanism of CO_2_ concentrations on typical industries, especially agriculture [81].

Scholars have utilized remote sensing and cloud computing to achieve a detailed characterization of agricultural land-use changes and obtain refined agricultural land-use data. This data is employed to optimize the crop parameterization schemes of land surface process models [82]. By coupling improved Organizing Carbon and Hydrology In Dynamic Ecosystems (ORCHIDEE) or Community Land Model (CLM) land surface process models with Weather Research and Forecasting Model (WRF) atmospheric model, scenario schemes are established to account for crop structure changes, such as “from water to drought” and “from drought to water,” as well as maturation changes, such as “double cropping to single cropping” and “single cropping to double cropping.” These schemes quantitatively evaluate the regional climate effects resulting from the macro-scale changes of cropland expansion in the north and reduction in the south [83]. Regarding bio-geophysical mechanisms, the influence of agricultural land use changes on surface radiation/energy balance is analyzed, and the extent to which these changes influence regional climate through alterations in radiation/energy components is determined and evaluated [[84], [85]]. In terms of bio-geochemical mechanisms, emphasis is placed on understanding the mechanisms by which agricultural land use changes affect regional climate through alterations in CO_2_ and CH_4_ emissions [86]. The Weather Research and Forecasting model coupled to Chemistry (WRF-Chem), considering atmospheric chemistry and transport processes, is employed to quantify the effects of agricultural land use changes on CO_2_ concentrations, thereby revealing the biogeochemical mechanisms through which agricultural land use changes affect regional climate by altering atmospheric CO_2_ and CH_4_ concentrations. Their research provides profound insights into the driving mechanisms of phenological changes on climate change and crop yield risks. Sun et al. [87] revealed that agricultural soil drilling increases the diffusion of gas, leading to reduce the CO_2_ concentrations in soil profile. This research contributes to understanding the mechanisms by which soil drilling affects agricultural carbon emissions.

In the context of rapid urbanization, the impact of land use transition on carbon emissions has garnered attention. Scholars such as Shi et al. [88] developed a theoretical framework that encompasses the interactions between climate change, land use change and the agricultural ecosystem within the context of global change and its effects on agriculture. They have revealed the interactions between climate change and land use changes, elucidating their quantitative effects on agricultural ecosystems. Additionally, they have proposed regional agricultural adaptation strategies in the context of global change [89]. Based on climate boundaries and land use boundaries, a method framework for quantitatively detecting the contributions of climate change to the transition of the agro-pastoral ecotone has been proposed. This framework has enabled the extraction of dynamic changes at a fine scale of 1 km, and the quantitative separation of contributions in terms of horizontal, vertical and boundary transitions. Shi et al. [88] also introduced a time-series superimposed method, utilizing the defined sequence and controlled sequence windows, to analyze the impacts of extreme climate events such as droughts and floods on crop yields. They have quantified the extent to which droughts and floods affect the production of main food crops. From both the qualitative and quantitative perspectives of land quality and quantity, they have systematically elucidated the influence of land use changes in the Huang-Huai-Hai Plain since 1990 on carbon emissions [90]. Roy et al. [91] have provided a comprehensive overview of the interactions and feedbacks mechanisms between urbanization and global environmental change, as well as the interactive decision-making processes and responses within and outside the land system to global environmental changes.

### 3.2 Agricultural land use carbon emission/reduction scenario simulation and analysis

A comprehensive assessment model for simulating and analyzing carbon emissions in agricultural land use, along with innovative approaches to optimize the allocation of agricultural land, can contribute to the practical realization of the carbon peaking and carbon neutrality goals. For spatial accounting and scenario simulation of carbon emissions in China and major countries in the world under 1.5 °C and 2 °C threshold temperature control scenarios, Ying et al. [92] conducted a study on temperature networks, CO_2_ networks and influencing parameters in major countries and typical regions in the world. They established a database of influencing parameters between the global surface temperature networks and CO_2_ network. Furthermore, a carbon emissions database was developed based on the Carbon Emissions Climate Monitoring Dataset, the Global Historical Climate Network Monthly Dataset (GHCN-V4) and the Global Climate Hazards and Risk Losses Database, allowing for calculations of carbon emissions and their contributions by major countries, economies or groups [93].

Lamb et al. (2022) analyzed the spatiotemporal variations in carbon emissions and their main driving factors in high-emission countries during 1970–2018. They estimated the spatial thresholds for carbon emissions under different temperature control scenarios [94]. Alcaraz et al. (2021) compared and analyzed the spatial distribution patterns of global historical per capita cumulative average carbon emissions, warming rates, and disaster loss rate. They generated an atmospheric CO_2_ emission-risk relationship graph and proposed a conceptual the concept of 'spatial threshold adjustment parameters for emission reduction' based on historical emission responsibilities and climate disaster risk [95]. Studies had already been conducted on the influence mechanisms of carbon emissions in system dynamics models. These studies have assessed the economic impacts of CO_2_ emissions on global temperature rise, developed the system dynamics model to measure carbon emission spatial thresholds, and incorporated modules on the impact of CO_2_ emissions, the impact of CO_2_ concentration on temperature, the regional economic response to temperature. Studies have also examined the cost-benefit analysis of carbon reduction, compared the costs of carbon reduction (RCC) and the incomes of carbon reduction (SCC), constructing efficiency indicators of carbon reduction (MEC) and examined the impact of global warming on labor losses [[88], [89],[96], [97]].

In terms of empirical research, Shi et al. [88] used a dataset of cropland and its changes from the 1970s to the 2000s to investigate the spatiotemporal variations in cropland caused by climate change in northern China since the 1970s, revealing the quantitative relationship between climate change, cropland quality and carbon emissions. Zhang et al. [98] published research on the mechanism through which climate change affects crop planting intensity, which found that the increase in cropped area and cropping intensity due to climate warming increased grain yield and carbon emissions to a certain extent.

Regional and functional differences in the land system have implications for carbon emissions per unit of GDP. These disparities, in turn, exacerbate regional differences in carbon emissions through resource circulation. By designing three scenarios of technological progress, the emission reduction effect of the land use system was explored and it was revealed that the reduction target can be effectively implemented by upgrading the existing infrastructure and strengthening the connectivity factor of the land system. Furthermore, Jin et al.[99] focused on the interconnect effects of future land use emission reduction on social and economic development, and answered the question of the impact of carbon emission reduction on the well-being of farmers' income . Taking Hubei Province in China as a case study, they extended the linear expenditure system model and the accounting method of carbon dioxide emissions, applied it to poverty alleviation and carbon dioxide emission reduction, and then introduced the decoupling analysis model to determine the relationship between carbon dioxide emissions and poverty levels, which provided valuable insights for the implementation plans of greenhouse gas control and socio-economic development arrangements. Additionally, Jiang et al. [100] pointed out that the spatial heterogeneity of carbon emissions affected global and even regional economies. Due to the uneven characteristics of carbon dioxide emissions, global temperature would rise, and temperature-sensitive economic sectors such as agriculture, mining and construction would suffer, of which southeast Asia, north-central Africa and northern South America would be hardest hit [100]. The publication of these findings further supports the empirical value and research significance of the agricultural land use carbon emissions/reductions scenario simulation and vision analysis.

### 3.3 Agricultural land use carbon emission/reduction mitigation and adaptation management

In the context of global climate change and rapid urbanization, a low-carbon economy has emerged as a fundamental pathway towards sustainable development. China's agricultural strategy for climate change mitigation and adaptation incorporates technological, policy, and practical approaches to reduce emissions and enhance productivity. Key mitigation efforts include promoting low-carbon practices, conservation tillage for soil carbon storage, and transforming agricultural waste into energy via biogas facilities. Additionally, afforestation efforts increase carbon sequestration. Adaptation strategies focus on developing resilient crop varieties and improving water efficiency with advanced irrigation. Initiatives like the North China Plain's water-saving projects and the “Grain for Green” program highlight significant improvements in water efficiency and the conversion of farmland into forests for increased carbon storage and biodiversity.

The targeted proposal to reduce carbon emissions intensity is crucial for formulating appropriate industry measures and achieving carbon emission reductions in land use. In addressing this research topic, Zhang et al. [101] develop a comprehensive quantitative framework based on refined indicators and a dynamic spatial panel model to assess the impacts of industrial structure and technological progress on carbon emission intensity across 281 prefecture-level cities in China from 2006 to 2016. The findings reveal significant positive spatial autocorrelation and heterogeneity of carbon emission intensity values in the land use system, with technological change and efficiency improvement emerging as the dominant factors driving changes in carbon emission intensity. However, the combined effects of optimizing industrial structure and technological progress in reducing carbon intensity are not as significant as initially expected [102]. Based on these insights, specific and targeted policy recommendations are proposed to reduce carbon emission intensity, including the promotion of regional green technologies, the integration of green technologies with green cities, the formulation of diverse urban development strategies, and the strengthening of regional cooperation [103]. Furthermore, Mohmmed et al. [104] reveal the relationship between CO_2_ emissions and economic development, as well as the driving factors, for the top ten carbon-emitting countries globally. They also evaluate the impacts and adaptation mechanisms of key sectors such as agriculture and energy on carbon emissions. Additionally, the book “Urbanization and Ecological Transformation: Theoretical Foundations, Methodologies, and Case Studies,” systematically outlines the theoretical implications and practical pathways for land use adaptation to climate change and the construction of an ecological civilization in the context of urbanization [105].

Regarding management measures for adapting agricultural land use carbon emissions and addressing climate change, researchers are not only focusing on the land system itself but also utilizing the life cycle theory. With the support of material flow analysis, they are revealing comprehensive and multi-chain adaptation measures for the production, transportation, and consumption stages of the land use system. Existing studies are particularly concerned about the significant social, ecological, and economic impacts of food waste in agricultural land use. They have conducted in-depth research on the current status, driving factors, and solutions to food waste. Based on large-scale field surveys conducted by the Ministry of Agriculture and Rural Affairs on food losses in the supply chain and by the Chinese Academy of Sciences on food waste in households and the catering industry between 2013 and 2018, Xue et al. (2021) employed material flow analysis, combined with literature data and text mining techniques, to systematically characterize the material flows of various food types in China's food supply chain [106]. They analyzed the scale and characteristics of food losses and waste, as well as their associated resource and environmental impacts. The study pointed out that within the ALUS, China's annual food losses and waste in terms of land, carbon, nitrogen emissions are equivalent to the consumption scale of a medium-sized country like the United Kingdom. This achievement has garnered wide attention from various sectors of society. The research emphasizes that to mitigate carbon emissions in land use, it is crucial not only to focus on agricultural land use itself but also to prioritize emission reduction management in other stages involved in agricultural land use. Only through such comprehensive efforts can the society achieve its carbon emission control objectives.

## 4 Resource and environmental carrying capacity as well as evaluation of agricultural land use system for food security

Assessment and regulation of the comprehensive effects of multiple components are key tasks in agricultural land-use system (ALUS) research. The ALUS is closely related to food security and the evaluation of resource carrying capacity [[107], [108], [109]]. The ultimate goal of detecting the spatiotemporal patterns and analyzing the processes and mechanisms of ALUS is to achieve an optimized balance between these systems and agricultural resources, environment, and ecology [[110], [111], [112]]. In the context of growing food and nutrition demands, this research serves the national food security strategy and the “dual carbon” goals. It aims to enhance agriculture's adaptability to climate change, ensure sustainable and efficient agricultural development, and clarify the coupling mechanism between food consumption patterns and resource-environment carrying capacity under changing national nutrition structures. Therefore, the section on resource security and environmental carrying capacity for food supply focuses on three main aspects: 1) Land–climate–water resources for food security, 2) Resource use efficiency of agricultural land use systems and food production, 3) Resource and environmental carrying capacity and food supply. These studies will enhance agriculture's adaptability to climate change, ensuring food security and the sustainable and efficient development of agriculture.

### 4.1 Land–climate–water resources for food security

Agriculture is one of the sectors most sensitive to climate change. Understanding the response of agricultural yield to climate change is crucial for timely, accurate, and effective agricultural adaptation of from a management perspective [113]. A series of studies this have been conducted to examine the interplay between land climate, water resources for food security using large-scale and long-term ground observation data [114]. A set of independent field control experiments were designed and conducted, which were integrated with research tools like spatial analysis, coupled crop mechanism model simulations, and mathematical methods such as Bayesian statistics. These collective efforts produced original findings that significantly contributed to the progress of scientific comprehension and methodological advancement.

Climate change will affect food production through changes in water, heat, and light resources. Although previous statistical and crop models have successfully isolated the effects of climate change on agricultural yield, fully understanding the overall impact of extreme climate events remains challenging. To directly assess the impact of extreme climate events on agricultural yield, a time-series overlay analysis method was developed. This involved constructing a spatial database of per-unit-area yields for major crops at the county level in China since 1982. The superposed epoch analysis (SEA) model was then employed to quantify crop yield and production losses due to droughts and floods across China [114]. Enhancing the understanding of cropland changes and their driving factors is a key focus for policy decision-makers in China. Datasets on cropland and cropland changes from the 1970s to the 2000s were utilized to investigate whether climate change has induced spatio-temporal changes in cropland in northern China since the 1970s [82]. These analyses indicate that climate change, along with socioeconomic factors, has been a driving force behind cropland changes in northern China over the past several decades. Databases containing maize yields and climate variables during the growing seasons were utilized to assess the vulnerability of African maize yields to climate change and variability. This assessment, conducted at the country level, considered different management levels over the period from 1961 to 2010 [115]. Overall, this research highlights the multifaceted impacts of climate change on agricultural productivity, emphasizing the necessity for targeted mitigation and adaptation strategies.

Understanding the factors influencing crop phenology is crucial for developing effective agricultural strategies in the face of climate change. Crop phenology is influenced by both climate change and crop management. However, attributing changes in crop phenology solely to climate change is challenging due to simultaneous changes in crop management practices. In China, the impacts of climate change and crop management on wheat phenology from 1981 to 2010 were isolated and quantified using a first-difference multiple regression model [116]. Although crop phenological changes and their responses to climate change, particularly temperature, have been investigated, the impact of agronomic practice such as cultivar shifts and planted date changes on crop phenology still requires investigation. A long-term dataset (1981–2010) of wheat phenology and corresponding local weather data from 48 agro-meteorological stations has been used to analyze phenological changes of spring and winter wheat [117]. Modeling results indicate that reductions in wheat growth duration due to climate change could be counterbalanced by introducing new cultivars with higher thermal requirements and accelerated by delaying the sowing date. These findings underscore the importance of adaptive agronomic practices in mitigating the effects of climate change on crop production.

Addressing the challenges posed by climate change is crucial for ensuring global food security and the sustainability of natural ecosystems. Despite the evident warming in China in recent decades, our current understanding does not enable a clear assessment of the impact of anthropogenic climate change on the country's water resources and agriculture, and consequently, on China's ability to feed its population [118]. Many regions that struggle with food security depend on local agricultural production to meet their nutritional needs. Predominantly located in tropical and subtropical areas, these regions are significantly impacted by global climate variations and fluctuations in global commodity prices [119]. As climate change intensifies, plant disease pressures are expected to significantly increase, thereby negatively impacting food security and the sustainability of natural ecosystems [120]. To address these challenges, a science–policy interface is needed to collaborate closely with relevant intergovernmental organizations. Such collaboration would provide effective monitoring and management of crop growth and disease under climate change, ensuring long-term food and nutrient security, as well as the sustainability of natural ecosystems.

### 4.2 Resource use efficiency of agricultural land use system and food production

To meet the decision-making needs for improving efficiency and optimizing resource utilization systems in rural revitalization, research was conducted to establish an index system and implement a multi-model analysis of resource utilization. A complementary model system, incorporating resource structure analysis, process inversion, pattern simulation, and effect evaluation, was developed with proprietary intellectual property rights. This system addresses the current technical requirements for agricultural factor allocation and resource efficiency assessment [121]. Additionally, efficiency evaluation methods have been proposed for assessing the ecological, technical, organizational, and system efficiency of rural revitalization. Climate change may affect these undernourished communities by reducing local yields and driving up global commodity prices due to significant decreases in the global production of corn, wheat, and rice [119]. Despite these challenges, the very low agricultural productivity in food-insecure countries presents a significant opportunity.

Achieving food security for all may be possible by transforming these agricultural systems with improved seeds, fertilizers, better land use practices, and enhanced governance. A study evaluated the tradeoffs between agricultural production and key ecosystem services, as well as their spatial distributions, at the watershed level in Zhangye using multisource observation data. Significant tradeoff relationships were observed between agricultural production and both water yield and soil conservation in the upper reach of Zhangye [122]. The findings indicated that increasing agricultural production would come at the cost of decreased water yield and soil conservation, particularly in the upper reach area. Quantifying and determining the spatial patterns of these tradeoffs is essential for developing regional ecological conservation policies and managing ecosystems sustainably.

With rapid economic growth and urbanization in China affecting agricultural land, improving eco-efficiency is crucial for sustainable agricultural development and ensuring food security. Using extensive natural and socioeconomic data, we estimated land productivity in Shandong, China, from 1990 to 2010 with the Estimation System of Land Production and analyzed eco-efficiency using Stochastic Frontier Analysis. The results showed that land productivity was unevenly distributed in Shandong, with relatively lower values in areas covered by urban development [123]. These findings indicate trade-offs between agricultural production and urbanization, highlighting the need to adjust agricultural technological measures based on local conditions to improve eco-efficiency and promote sustainable agricultural development. A systems-based perspective connects food security to agricultural productivity, food safety, health and nutrition, as well as processing and supply chain efficiency, in the context of global and industry megatrends [124]. A collaborative, transdisciplinary approach to food security science, focusing on enabling technologies within the framework of social, market, and global trends, is essential to achieving food and nutritional security.

To meet the Sustainable Development Goals for the water–food–energy–ecosystems nexus, integrated assessments are essential for measuring the impact of global change on natural resources. The Global Biosphere Management Model was utilized, incorporating constraints from water availability, environmental flow requirements, and water use across agriculture, industry, and households, simulated using the Lund–Potsdam–Jena managed Land model, the Environmental Policy Integrated Climate model, and the WaterGap model [125]. The findings indicate that an additional 100 million hectares of land would be needed to double food production by 2050 to meet projected food demands. Additionally, international trade would need to nearly triple to satisfy future crop demands, with an extra 10–20% of trade flow required from water-abundant regions to water-scarce regions to maintain global environmental flow requirements. Potential climate-related impacts on future crop yields are a major societal concern. While future yield estimates remain uncertain, these findings suggest that major breadbasket regions will [126]. Afforestation, often modeled by imposing carbon prices on land carbon stocks, could significantly impact food security compared to non-CO_2_ emissions policies, which are generally implemented as emissions taxes [108]. This underscores the need for better coordination between emissions reduction and agricultural market management policies, as well as improved representation of land use and associated greenhouse gas emissions in models.

### 4.3 Resource and environmental carrying capacity and food supply

Addressing food loss and waste is vital for achieving sustainable food systems and long-term food security. The material flow analysis was used to systematically characterize the material flow of primary food types in China's food supply chain. This analysis examined the scale and characteristics of food loss and waste (FLW), as well as their associated resource and environmental impacts. Reducing FLW is crucial for ensuring food security. Quantifying FLW for major agrifood products across the entire farm-to-fork chain in China was accomplished using six years of large-scale field surveys and data extracted from existing literature [106]. The analyses showed that 27% of the food annually produced for human consumption in the country (349 ± 4 Mt) is lost or wasted, 45% of which is associated with postharvest handling and storage, and 13% with out-of-home consumption activities. To ensure long-term food security, it is essential to operate within the limits of available resources and maintain environmental health and integrity. Sustainable land management practices, such as conservation agriculture, agroforestry, and land-use planning, help optimize land use while preserving its capacity for food production.

To achieve food security, it is crucial to use land efficiently and avoid practices that lead to degradation, such as deforestation, overgrazing, or excessive use of agrochemicals [127]. Protected areas (PAs) are vital for biodiversity conservation but are threatened by cropland expansion. The dynamics of cropland within PAs reveal that this expansion is a persistent global conservation challenge, which threatens the goals of the post-2020 global biodiversity framework [128]. Cropland where food and feed are grown is the central and limiting resource for food production. In most regions, diets have become richer, while the land required to feed one person has decreased. In many areas, dietary changes may soon surpass population growth as the primary driver of land requirements for food [127]. Some findings support an approach where producers monitor their own impacts, flexibly meet environmental targets by choosing from various practices, and communicate their impacts to consumers [129]. The scale of the human population and its current growth rate significantly contribute to the loss of biological diversity [130]. Preserving biodiversity and ecosystem health is essential for maintaining the long-term carrying capacity of these services.

China's increasingly urbanized and affluent population is driving a growing and evolving demand for food, which may not be met without a significant increase in agricultural productivity and the sustainable use of natural resources. The push for higher food production has significantly impacted the environment, with the deterioration of ecosystem quality due to historic and current pollution levels potentially compromising China's food production system. Addressing the grand challenges involves not only increasing food production but also doing so sustainably, without causing environmental degradation [131]. Estimates of China's future food trade patterns and associated water transfers have been made, quantifying the effects of targeted reductions in irrigated land on water consumption and food self-sufficiency. This analysis also accounts for production displacement and local water productivity [132]. As socioeconomic growth and the associated pressure on water resources continue to increase, it is essential to view food production as an integral part of the environmental system, encompassing soil, air, water, and biodiversity, rather than treating it as separate from it.

There is a significant relationship between food loss and carbon dioxide emissions. The direct relationship between food loss and CO_2_ emissions is primarily through the unnecessary consumption of resources in the production, transportation, and storage of lost food. A study in the United States revealed that food loss accounts for 28% of the carbon footprint of the average diet, equivalent to the annual emissions of 33 million passenger cars. Particularly, beef loss, though only accounting for 4% of the retail food supply, contributes 36% of diet-related greenhouse gas emissions. This is due to the high emissions associated with beef production compared to other foods. Globally, production-phase greenhouse gas emissions from food loss and waste have tripled over the past 50 years, reaching 220 million tons of CO_2_ equivalent annually. This indicates the critical need to improve food supply chain efficiency to reduce emissions as global population and food demand increase [132]. Moreover, reducing food loss not only increases the amount of food available for consumption but also significantly reduces greenhouse gas emissions associated with food production, thereby mitigating climate change impacts [133]. For example, reducing food loss in developed countries can decrease the consumption of resources such as land, water, and fossil fuels, and lower greenhouse gas emissions [134]. Therefore, reducing food loss is a crucial measure for achieving sustainable food systems and reducing environmental burdens.

Improving food security is often accompanied by increased carbon dioxide emissions. A study in Sub-Saharan Africa found that CO_2_ emissions positively impact food availability and accessibility but have no significant effect on food utilization. The study also showed a short-term causal relationship between food availability and CO_2_ emissions, indicating that increased CO_2_ emissions can promote food availability in the short term. In developing countries, improving food security significantly increases CO_2_ emissions. Research indicates that for every 1% increase in food security, CO_2_ levels rise by 32%. This suggests that improvements in food security, driven by economic and social development, are often accompanied by increased agricultural production and energy consumption, leading to higher CO_2_ emission. Additionally, global research shows a significant relationship between agricultural activities and CO_2_ emissions. Agricultural production, including crop cultivation and livestock farming, generates substantial greenhouse gas emissions, negatively impacting the environment. To address this challenge, it is essential to adopt measures that reduce CO_2_ emissions in agricultural production while enhancing food security. Examples include promoting low-carbon agricultural technologies, optimizing energy use structures, and improving agricultural production efficiency [135]. Balancing food security and CO_2_ emissions is thus a critical challenge in addressing climate change and ensuring food security.

Climate-smart agriculture (CSA) is an approach designed to transform and reorient agricultural systems to ensure food security amidst the challenges of climate change. Widespread changes in rainfall and temperature patterns threaten agricultural production and increase the vulnerability of those who depend on agriculture for their livelihoods, including much of the world's poor. Climate change disrupts food markets, posing risks to the food supply on a population-wide scale. These threats can be mitigated by enhancing farmers' adaptive capacity, resilience, and resource use efficiency in agricultural production systems. CSA promotes coordinated actions among farmers, researchers, the private sector, civil society, and policymakers towards climate-resilient pathways through four main action areas: (1) building evidence; (2) increasing local institutional effectiveness; (3) fostering coherence between climate and agricultural policies; and (4) linking climate and agricultural financing [136]. Unlike 'business-as-usual' approaches, CSA emphasizes the implementation of flexible, context-specific solutions, supported by innovative policy and financing actions.

## 5 Layout and optimization of agricultural land use capacity enhancement

In the new development stage, the combination of the data-driven techniques and ALUS management has the potential to revolutionize various aspects of agricultural land use, including forecasting, monitoring, decision-making, and optimization, providing new solutions for the low-carbon, green, and efficient development of ALUS. An important trend in agricultural land use is the application of deep learning and machine learning technologies. Deep learning algorithms, including Convolutional Neural Networks (CNN), have demonstrated significant potential in diverse agricultural applications, such as predicting crop yield, detecting diseases, and identifying weeds [137]. Another trend involves the utilization of big data analysis technology for managing ALUS. Big data analysis technology enhances the usability, interpretability, and discoverability of multi-source, multi-scale, and heterogeneous datasets, including satellite imagery, weather data, and sensor data. This facilitates more accurate and efficient analysis and decision-making for ALUS [138]. Moreover, the application of artificial intelligence (AI) and machine learning technologies in precision agriculture has garnered significant attention. Precision agriculture employs technologies such as sensors, drones, and AI algorithms to collect and analyze data on soil conditions, weather patterns, crop health, and other factors. This data-driven approach assists agricultural producers in determining optimal locations for various crop and livestock production, optimizing the usage of resources such as water, fertilizers, and pesticides, as well as maximizing productivity, profitability, and sustainability. As a result, it optimizes resource allocation and enhances agricultural practices [139]. These trends hold the potential to fundamentally transform the management of agricultural land use and foster more efficient, sustainable, and productive agricultural systems.

To further explore the innovative approach of the ALUS in coordinating the implementation of the “dual carbon” goals and ensuring food security and to improve optimal ALUS management and build its capacity, it is essential to support the development of new technologies, models, and system platforms that leverage big data. By addressing these challenges and leveraging these trends, the agricultural sector can benefit from increased productivity, sustainability, and resilience.

### 5.1 Research and development of three-dimensional monitoring and data fusion technology for the alus

ALUS generate vast amounts of data from various sources such as remote sensing, sensors, and farm management systems. However, the complexity of agricultural land changes and the limitations of traditional methods of analysis have hindered the understanding of multidimensional information related to the ALUS. Ensuring data interoperability, standardization, and security are crucial for effective utilization of AI and big data technologies in ALUS management [[140], [141]]. The fourth big-data-driven paradigm provides new opportunities for research on optimal ALUS management. Multi-source remote-sensing data, ground-based networked observation data, and crowdsourced big data form a data acquisition channel for three-dimensional monitoring, providing valuable information on resource allocation, vegetation health, carbon dynamics, water resources, and other important parameters for effective land use planning and decision-making.

Some fields have benefited from three-dimensional monitoring and data fusion for ALUS management under the fourth big data-driven paradigm. These benefits include: (1) Improved understanding of changes in agricultural land use. Big data analytics can assist researchers in accurately mapping and monitoring changes in land use at various scales, including global, regional, and local levels. This provides valuable information regarding the drivers and patterns of land use changes, enabling improved management strategies [142]. (2) Optimization of resource allocation. Big data analytics can optimize the allocation of resources such as water, fertilizers, and pesticides in ALUS. By analyzing data on soil conditions, weather patterns, and crop health, AI algorithms can provide recommendations for efficient resource management, leading to improved productivity and reduced environmental impact [143]. (3) Integration of ecosystem services: The use of big data and AI can facilitate the integration of ecosystem services into decision-making processes. By quantifying and valuing the ecosystem services provided by agricultural land, such as carbon sequestration, water regulation, and biodiversity conservation, policymakers and land managers can make more informed and sustainable land use decisions [144]. (4) Identification of socio-economic drivers. Big data analytics can help identify and analyze the socio-economic drivers that influence land use decisions. By examining data on population, wealth, consumption preferences, and trade, researchers can gain insights into the factors that shape land use patterns and trends [145]. (5) Assessment of sustainability and resilience. Big data analytics can contribute to the assessment of the sustainability and resilience of ALUS. By analyzing data on greenhouse gas emissions, water use efficiency, and other sustainability indicators, researchers can evaluate the environmental and socio-economic impacts of different land use practices and identify opportunities for improvement [146].

Despite these opportunities and benefits, there are also several technical difficulties and research trends that need to be addressed. Some of the main technical difficulties include data availability and quality, model development, infrastructure, and technical expertise. Research trends in the field include the use of remote sensing technologies, precision agriculture, integration of emerging technologies (e.g., internet of things and blockchain technology), and the development of decision support systems.

### 5.2 Model integration innovation for policy and management simulation analysis of agricultural land use systems

The integration of model-based analysis and simulation in ALUS policy and management research offers innovative approaches to understanding and optimizing land use decisions. By considering multiple dimensions, evaluating trade-offs, and assessing the impacts of different policies and technologies, integrated models can inform decision-making processes, enhance sustainability, and support the transition towards more efficient and resilient ALUS.

Model integration offers numerous key benefits in the management of agricultural land use systems. Firstly, it enables the assessment of various land use scenarios and their potential impacts on multiple dimensions, including food production, environmental sustainability, and socio-economic factors. This allows policymakers and land managers to gain a better understanding of the trade-offs associated with different policy decisions and management strategies [145]. Secondly, model integration provides an analytical framework to evaluate the effectiveness of policy interventions and management practices. By simulating the outcomes of different scenarios, decision-makers can make informed choices that optimize agricultural land use efficiency, productivity, and sustainability [147]. Moreover, integrated models can identify potential synergies and co-benefits across different sectors and sub-objectives. They can also assess the potential impacts of technological advancements and innovation on improving productivity, resource efficiency, and environmental performance of ALUS [148]. Additionally, model integration allows for the consideration of land use dynamics and management strategies at various scales, ranging from local to regional and global. This enables scholars to analyze the interactions between different land use systems, evaluate the implications of land use changes across scales, and inform policy coordination and spatial planning. Lastly, integrated models can evaluate the impacts of climate change on ALUS and assess the resilience and adaptive capacity of different management strategies [149].

For example, in China, Deng (2011) has built a platform for land use policy and management simulation and analysis, including the CGELUC, DLS, and ESAP models, to simulate land use policy and management and to provide decision support for the sustainable use of regional agricultural land resources [29]. The DLS model is based on the equilibrium theory of regional land structure change and the theory of land type distribution constraints at the raster scale. It considers the influence of climatic and human factors, quantitatively analyzes the dynamic feedback mechanism, and jointly promotes the mechanism of land system structure change and pattern evolution. The ESAP includes modules for land resource stock estimation, land use type and intensity determination, and crop growth demand for light, temperature, water, and other climatic resources. Different levels of crop productivity were estimated, namely, photosynthetic production potential, light-temperature production potential, climate production potential, land production potential, and cropland productivity. The DLS and CGELUC models were applied by the Ministry of Water Resources, Ministry of Natural Resources, and relevant provincial departments, respectively.

### 5.3 Construction of an alus management platform driven by big data and cloud computing

The integration of big data technology and cloud computing presents numerous opportunities for influencing and improving the management of agricultural land use. In particular, big data is transforming traditional ALUS by introducing innovative approaches to decision-making and resource allocation. This paradigm shift, characterized by data-driven decision-making, reflects a broader trend in management sciences where big data technologies are fundamentally altering traditional approaches [150]. By leveraging big data, ALUS can be optimized through more precise and informed decision-making processes, leading to improved sustainability, productivity, and resilience. These innovations in management practices are part of a broader shift towards data-centric decision-making frameworks that are increasingly essential for sustainable land-use management [151]. Key advancements include On-Farm Experimentation (OFE), rapid data processing, precision agriculture, effective data integration, monitoring of land use changes, integration of multiple factors and scales, and improved ecosystem monitoring and conservation.

Several potential innovative approaches can be highlighted. Firstly, the combination of OFE with big data and digital technologies can support research on agroecological landscapes by informing the integration of analytical scales. This integration can help in understanding the impacts of different land use practices on ecosystem dynamics and inform management decisions [152]. Secondly, rapid data processing and accessibility, along with effective searching, retrieving, and integration of data, are essential as more high-resolution data becomes available. Big data tools, such as distributed databases, massively parallel processing, and cloud computing, enable researchers to search, retrieve, analyze, and integrate data effectively for land use management research [153]. Platforms like Google Earth Engine facilitate the analysis of big data, leading to the development of high-resolution land use and land cover products with unprecedented spatiotemporal resolutions [154]. Thirdly, precision agriculture and the optimization of resource use, enabled by big data and cloud computing, allow for the targeted application of inputs like water, nutrients, and pesticides only where and when they are needed. This optimization enhances agricultural efficiency, reduces environmental impact, and improves productivity [155]. Additionally, agricultural land use change monitoring and assessment are crucial at various scales. By integrating data from various sources, including remote sensing, citizen science databases, and ecological models, researchers can assess the impacts of land use change on biodiversity, ecosystem services, and environmental health. This information then informs conservation efforts, providing valuable insights for managing ALUS.

The ALUS management platform employs big data analytics and cloud computing to facilitate the transformation of agricultural land management. The integration of diverse data sources, including satellite imagery, soil metrics, weather patterns, and crop yield data, facilitates informed decision-making, enhances productivity, and promotes environmental conservation. The application of big data analytics to agriculture enables precision techniques to be employed, which in turn allows farmers to optimize crop rotations, implement targeted irrigation, reduce the use of fertilizers and pesticides, and increase yields while minimizing the environmental impact. This approach ensures the efficient use of resources and the conservation of ecosystems. Cloud computing ensures the platform's scalability and accessibility, thereby enabling users worldwide to access vital information for effective land-use planning. The democratization of information facilitates collaborative sustainable agriculture efforts.

The integration of big data and cloud computing in ALUS is aligned with the Sustainable Development Goals (SDGs), particularly SDG 2 (Zero Hunger), SDG 12 (Responsible Consumption and Production), and SDG 15 (Life on Land). The platform optimizes production, reduces waste, and supports biodiversity and land conservation. Pilot projects have demonstrated the potential of the platform. In Brazil, the utilization of satellite data and analytics has facilitated a reduction in Amazon deforestation. In Kenya, the implementation of cloud-based platforms has enabled the delivery of precision farming recommendations, resulting in enhanced yields and a reduction in water usage. Nevertheless, several challenges remain, including data privacy, the existence of digital divides in rural areas, and the need for technical skills. For solutions to be implemented, it is necessary to establish data protection policies, develop digital infrastructure, and create training programmes to enhance digital literacy among farmers. In conclusion, the ALUS platform employs the use of big data and cloud computing to advance sustainable agriculture. This is achieved by balancing productivity with environmental conservation, thereby contributing to the achievement of SDGs.

In conclusion, the utilization of these innovations enables stakeholders to employ high-precision mapping and early monitoring of various crops. This approach elucidates the mechanisms underlying climate effects and addresses the knowledge gap regarding the attribution of interannual fluctuations in grain yield. Furthermore, it encourages the convergence and advancement of management science, remote sensing science, land system science, and earth system science with AI. Furthermore, stakeholders can continue to provide system and platform support for the innovative management of ALUS.

## 6 Summary and future perspectives

**Fig. 2:**
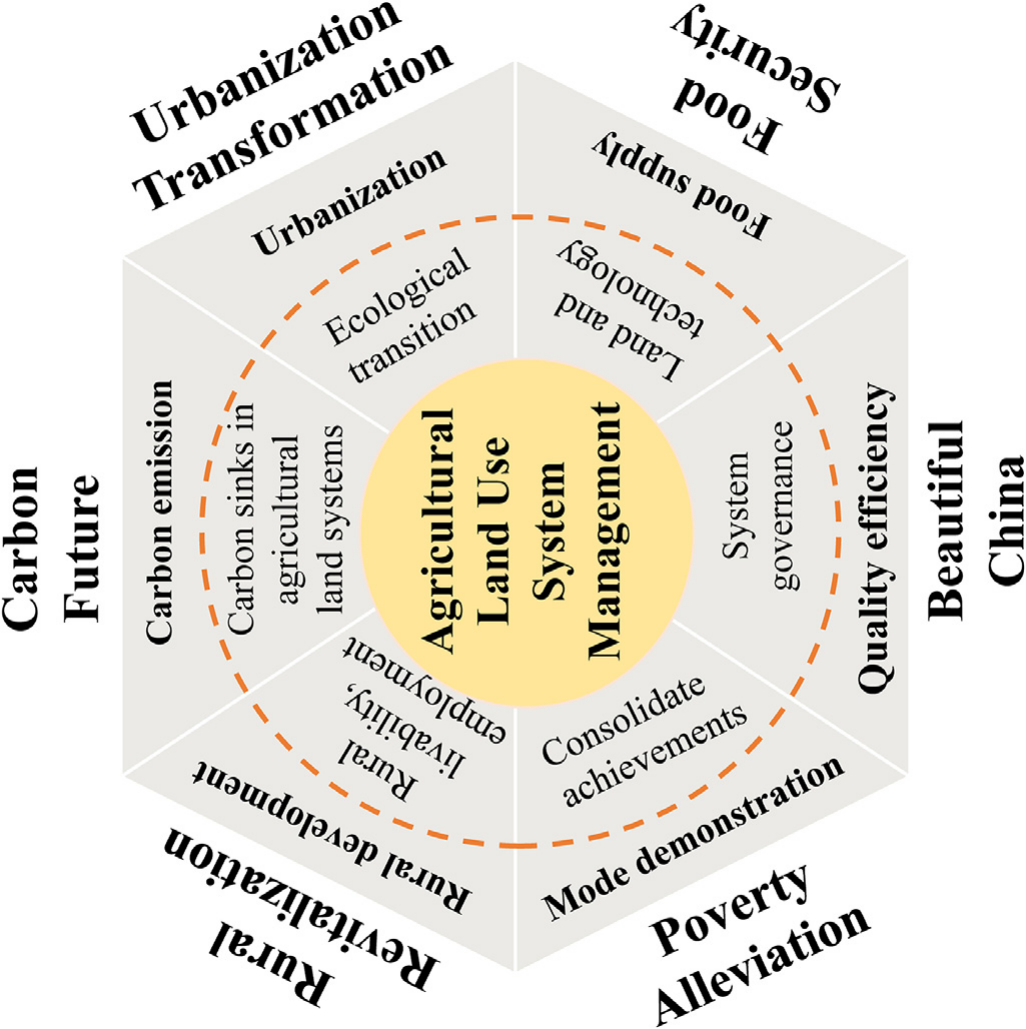
Schematic diagram of agricultural land use system management for future carbon emission, urbanization, food security, rural revitalization, poverty alleviation, and a beautiful China.

Based on the research strengths and comparative advantages formed during long-term evolution, the specific research objective is the development of a theoretical system and governance mechanism for the management of the ALUS while working toward the “dual carbon” goals, urbanization, food security, rural revitalization, poverty alleviation, and a beautiful China (Fig. 2). The focus will be on constructing a theoretical system, conducting applied research and empirical studies, and building a systematic platform for analyzing the complexities and problems of ALUS, which clarify the management ideas and framework for the system, and create a “trinity system” research framework and systematic platform that integrates basic theory, applied research, and decision support. Later research should emphasize the basic theory, guidance on major problems, and support for key policies. The planning is getting on track, which is developing a relevant theoretical ALUS management system and governance mechanism with Chinese characteristics. This goal is consistent with the existing research foundation. Over the years, rich research results and experience were accumulated in multiple foundational fields related to ALUS management, such as spatiotemporal pattern simulation analysis, big data information theory, resource optimization and allocation simulation, system coupling analysis, climate change response simulation, sustainable development, and governance. This lays a solid foundation for the group to achieve its research goals.

### 6.1 Key scientific issues

Based on practical needs in ALUS management, assessing land use will continue to focus on the scientific forefront of the theoretical ability of ALUS management to reduce carbon and other greenhouse gas emissions and ensure food security. The aim is to develop an ALUS decision support platform based on artificial intelligence plus (AI+) and a theoretical system and management practices for an ALUS that meets the socioeconomic, ecological, and cultural needs of China (called “with Chinese characteristics”). Through five years of study, following four research questions are hoped to answer.

How can future research construct an ALUS theory with Chinese characteristics by combining regional characteristic induction and general law cognition? What are the driving forces and mechanisms of the evolution of an ALUS dominated by agricultural development laws with Chinese characteristics? How can future research construct a theoretical system for an ALUS with Chinese characteristics by combining the induction of regional characteristics and universal law cognition?

What are the potential changes in agricultural carbon and other greenhouse gas emissions in China? What is the mechanism and effectiveness of regulating greenhouse gas emission reduction from point, line, and surface on multiple scales based on the ALUS?

How can government plan a resource-guarantee strategy for China's food security against a background of unprecedented changes? How can the regional resource distribution of food production capacity in the ALUS be adjusted to meet the demand for dietary structure changes in China?

What are the goals and orientations of the regional agricultural transformation and development driven by AI and the digital economy? How can the quality, efficiency, and optimization management of ALUS be arranged?

### 6.2 Future research perspectives

**Fig. 3:**
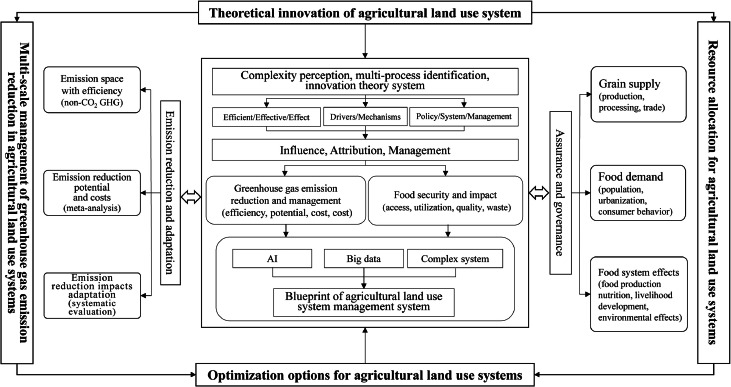
**Prospects for future research directions in the field on ALUS management**.

**Fig. 4:**
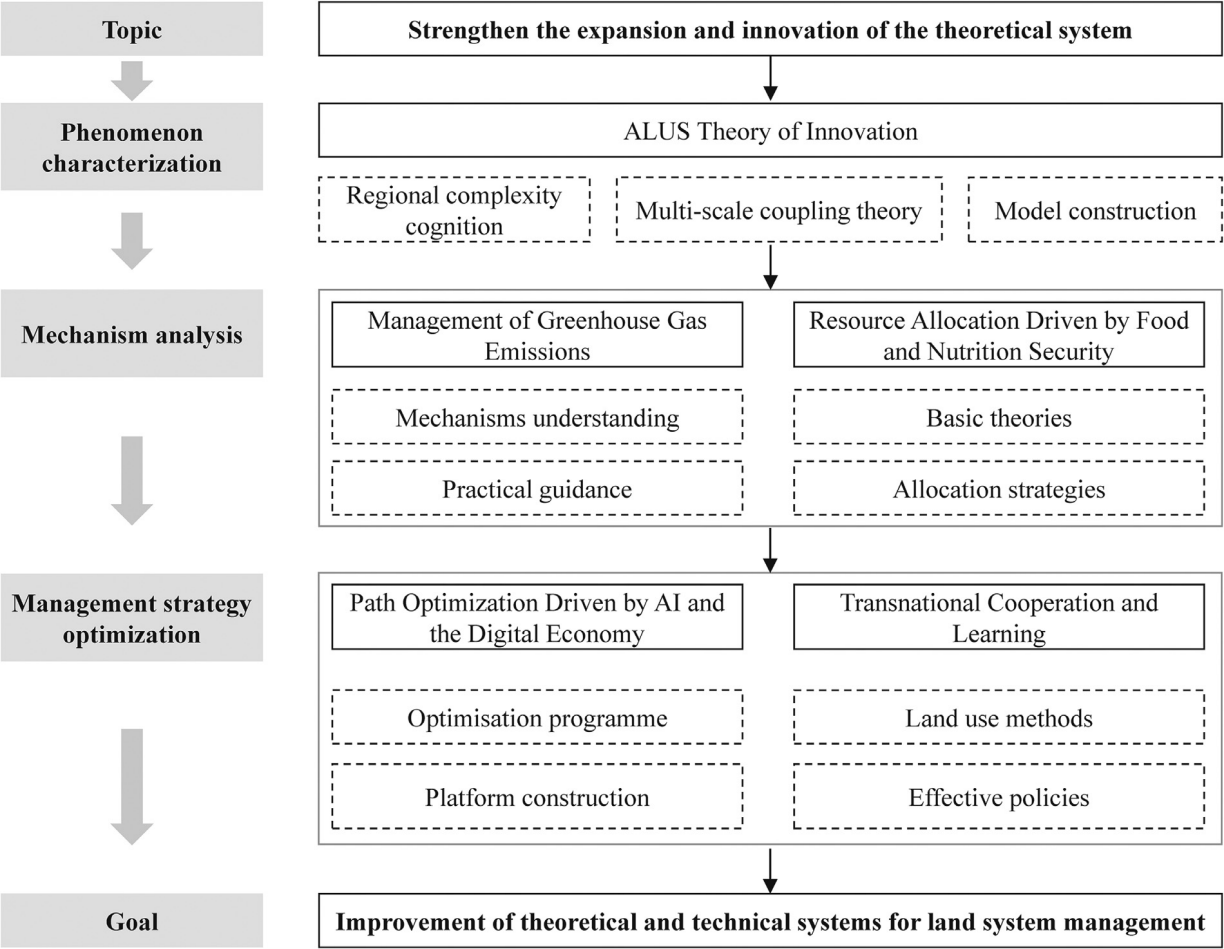
**Future expansion of the theoretical system of the ALUS management system**.

The aim is to strengthen the expansion and innovation of the theoretical system and focus its research on management practices in four major directions: theoretical innovation of the ALUS under the guidance of carbon emission reduction and food security, multi-scale comprehensive management of greenhouse gas emission reduction in the ALUS, resource allocation driven by ALUS food and nutrition security, and path optimization of agricultural transformation and development driven by artificial intelligence and the digital economy. The four major research directions shown in Fig. 3, Fig. 4, from phenomenon characterization and mechanism analysis to management strategy optimization, are ultimately linked with each other. The aim of the ALUS is to promote the refinement and improvement of relevant theoretical and technical systems for the management of land systems, and to improve policymaking and practice under the objectives of carbon neutrality, peak emissions and food security, by accelerating interdisciplinary construction with ALUS at its core. The research directions are as follows:

I. Theoretical innovation in the ALUS under the guidance of carbon emission reduction and food security. Based on clarifying the general laws of the coupled relationships among subsystems such as land use, climate change, and food security in the ALUS, a comprehensive study on the integration of the theoretical system and governance mechanisms for ALUS management towards low-carbon and high-quality development with Chinese characteristics will be conducted. Over five years of future research, it is intended to fully elucidate the multiscale, dimensional and regional feedback mechanisms within and between the subsystems of ALUS, and to develop a comprehensive coupled theoretical system and analysis framework. Therefore, this research includes two aspects: regional ALUS complexity cognition and mechanism identification as well as complex multiscale coupling theory innovation and model construction of regional ALUS.
II. Comprehensive management research on greenhouse gas emission reduction in the ALUS. Research on greenhouse gas emissions and management from the perspective of the ALUS can deepen understanding of the impact mechanisms of regional carbon cycling and can comprehensively guide low-carbon development of the economy and society in fields such as land system planning, industrial structure adjustment, land development and remediation, and urban–rural construction.
III. Resource allocation in the ALUS driven by food and nutrition security. Through research over the next five years, the basic theories and main measures of the resource and environmental carrying capacities and resource allocation strategies of the ALUS under nutritional needs and food security will be more comprehensively revealed.
IV. Optimization schemes for the ALUS driven by AI and the digital economy. Through research over the next five years, the technical realization and optimization upgrade schemes of AI+ supporting ALUS management in the digital economy will be more comprehensively formed. The construction of a decision support platform for ALUS management will be pursued, driven by artificial intelligence and digital economy optimization schemes.
V. Transnational cooperation and learning for better farmland management. International studies help us learn from others, find our gaps, and innovate. Research can improve land use planning, efficiency, sustainability, ecological protection, rural revitalization, and food security. By comparing countries, we find effective methods and policies for efficient land use, ecological balance, rural growth, and food security strategies.

## Declaration of competing interest

The authors declare that they have no conflicts of interest in this work.
